# The Age-Related Changes in Speed of Visual Perception, Visual Verbal and Visuomotor Performance, and Nonverbal Intelligence During Early School Years

**DOI:** 10.3389/fnhum.2021.667612

**Published:** 2021-08-17

**Authors:** Rana J. Alghamdi, Melanie J. Murphy, Nahal Goharpey, Sheila G. Crewther

**Affiliations:** ^1^Department of Psychology and Counselling, La Trobe University, Melbourne, VIC, Australia; ^2^Department of Psychology, King Saud University, Riyadh, Saudi Arabia

**Keywords:** sensory processing speed (PS), young school-age children, visual inspection time, visual verbal RAN processing, SLURP visuomotor processing, Raven’s Nonverbal intelligences

## Abstract

Speed of sensory information processing has long been recognized as an important characteristic of global intelligence, though few studies have concurrently investigated the contribution of different types of information processing to nonverbal IQ in children, nor looked at whether chronological age vs. months of early schooling plays a larger role. Thus, this study investigated the speed of visual information processing in three tasks including a simple visual inspection time (IT) task, a visual-verbal processing task using Rapid Automatic Naming (RAN) of objects as an accepted preschool predictor of reading, and a visuomotor processing task using a game-like iPad application, (the “SLURP” task) that requires writing like skills, in association with nonverbal IQ (Raven’s Coloured Progressive Matrices) in children (*n* = 100) aged 5–7 years old. Our results indicate that the rate and accuracy of information processing for all three tasks develop with age, but that only RAN and SLURP rates show significant improvement with years of schooling. RAN and SLURP also correlated significantly with nonverbal IQ scores, but not with IT. Regression analyses demonstrate that months of formal schooling provide additional contributions to the speed of dual-task visual-verbal (RAN) and visuomotor performance and Raven’s scores supporting the domain-specific hypothesis of processing speed development for specific skills as they contribute to global measures such as nonverbal IQ. Finally, RAN and SLURP are likely to be useful measures for the early identification of young children with lower intelligence and potentially poor reading.

## Introduction

Significant correlations between measures of speed of information-processing (inspection time and reaction time tasks) and intelligence were first described in young adults more than 40 years ago by Vickers et al. (1972) and others later on (Jensen and Munro, 1979; Vernon, 1983; Nettelbeck et al., 1986; Deary et al., 2001; Grudnik and Kranzler, 2001; Jensen, 2006, 2011; Sheppard and Vernon, 2008). Around this time brain imaging techniques were appearing and Positron Emission Tomography (PET) was used to demonstrate that smarter adult brains work more efficiently with faster rates of information processing and utilize less neural energy than individuals with lower IQ scores (Haier et al., 1988, 1992; Jung and Haier, 2007).

Since then the speed of visual information processing has been investigated using a variety of paradigms including simple sensory perceptual tasks such as visual inspection time tasks, more complex coding tasks, and dual response time tasks (Blake, 1974; Kail and Park, 1992; Miller and Vernon, 1997; Weiler et al., 2003; McAuley and White, 2011). In particular, processing speed in children has been shown to increase with chronological age (Case, 1985; Anderson, 1996; Anderson et al., 1997). Indeed, in a review of 72 published studies, Kail (1991a) found that motor reaction times of young children (4–5 years) to visual stimuli were a third of the rate of adults, whereas older children (8 years) performed only twice as slowly as adults, raising the questions of whether: (i) these age-based changes in processing time were predominantly cognitive or motor-based development; (ii) whether the improved speed was more aligned to chronological age or domain-specific school routine based changes (Chi, 1977; Logan, 1988), and (iii) to what extent chronological age or months attending formal schooling (MAS), the related increase in the rate of visual processing mediates non-verbal IQ.

Thus, this study aimed to investigate rates of visual processing and whether differences in visual cognitive processing speed were due to individual school type experiences learning to read and write (domain-specific knowledge) or a single, global mechanism such as fluid nonverbal IQ that drives the exponential rate of visual information processing speed during child development (Kail and Park, 1992). To do this, we concurrently assessed the contribution of rates of simple, non-motor measured visual object recognition (Inspection Time task) modified from Vickers et al. (1972), visuo-verbal information processing (Rapid Automatic Naming of objects), and visuomotor eye-hand co-ordination and age and schooling to the prediction of non-verbal IQ (a global mechanism). The RAN of familiar objects task was chosen both as a measure of visual object verbalization and because it is a well-accepted predictor of future reading ability (Denckla and Rudel, 1974; Crewther et al., 2011, 2017; Siddaiah and Padakannaya, 2015; Savage et al., 2018; Landerl et al., 2019; Peters et al., 2020). Visuomotor skills have traditionally been assessed in terms of complex tasks with emphasis on manual timing (Tiffin and Asher, 1948; Wilson et al., 2000; Hart et al., 2006), rather than as measures of speed of accurate eye-hand coordination. Hence, the current study assessed visuomotor performance by sensitively recording the time taken and errors made in tracing five prescribed shapes in an iPad app known as the SLURP task (SLURP; Lee et al., 2014). Age-related contributions to nonverbal IQ were also measured using the raw scores on the Raven’s Colour Progressive Matrices (RCPM) nonverbal measure of reasoning ability, rather than standard scores that are corrected for developmental changes (Fry and Hale, 2000) and hence, likely to confound investigations of the age–related differences in multiple age groups. We expected that months of formal schooling would independently contribute to the development of the visual-verbal processing and visuomotor skills required for early school year foci of reading and writing (Burrage et al., 2008; Brod et al., 2017; Morrison et al., 2019), rather than simple visual perception, in line with the domain-specific knowledge hypothesis.

## Materials and Methods

### Participants

One hundred primary school beginners (Males = 49 and Females = 51; Prep *n* = 57, Grade 1 *n* = 28, and Grade 2 *n* = 15), and three age groups; 5 years (*n* = 31), 6 years (*n* = 39) and 7 years (*n* = 30) were recruited from three primary schools in metropolitan Melbourne, Australia (see Table 1). Parents/guardians received a written description of the research tasks and were informed that they could withdraw their child from the study at any stage as per the Declaration of Helsinki. Parents/guardians provided written consent for their child to take part in the study and verbal consent to participate in the study was also obtained from children prior to the commencement of testing sessions. This study was conducted with approval from the La Trobe University Human Ethics Committee, the Victorian Department of Education Human Ethics Committee, and the Victorian Catholic Schools Ethics Committee (HEC 18139). Inclusion criteria required adequate vision and hearing, neurotypical development, and age appropriate English-speaking ability.

**Table 1:** Mean (SD) and range for chronological age (years), Grade and nonverbal IQ (RPCM) raw scores for each age group.

Age group	*N*	*M* (SD)	IQ	(SD)	Range
5 years old	31	5.62 (0.22)	18.37	3.64	13–18
6 years old	39	6.38 (0.28)	20.54	4.54	12–30
7 years old	30	7.48 (0.33)	26.37	3.88	17–32
Prep	57	5.91 (0.38)	19.18	4.08	12–30
Grade 1	28	6.81 (0.40)	24.89	4.53	17–32
Grade 2	15	7.68 (0.26)	26.50	3.98	20–32

### Materials

#### Screening Measures

##### Nonverbal Intelligence

Nonverbal IQ was measured using the (Raven’s, 1958, 1995) Coloured Progressive Matrices (RCPM) a well-normed culture, and language-free psychometric test of non-verbal reasoning (Raven et al., 1998; Cotton et al., 2005). The RCPM consists of 36 colored matrices. Each matrix has one piece missing, and participants are asked to choose the most appropriate missing piece from six possible options. This task was developed for individuals between 5–11 years old and presents with high-reliability *r* = 0.80 (Raven et al., 1998; Cotton et al., 2005).

#### Experimental Measures

##### Inspection Time (IT) as a Simple Measure of Visual Object Recognition

**Figure 1:**
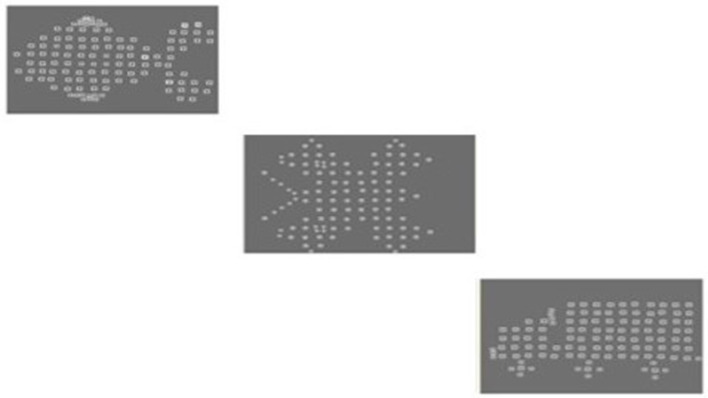
The fish, butterfly, truck (FBT) Inspection Time task consisting of three images rapidly and randomly presented.

A non-motor IT task modified from Vickers et al. (1972) by Brown and Crewther (2017) and Ebaid and Crewther (2019) was used to assess visual information processing and visual attention. This task is a simple computerized measure of the minimum time required to identify one of three simple stimuli flashing on the screen (Fish, Truck, or Butterfly) using a PEST (parametric estimation of statistical threshold) routine (see Figure 1). Following the presentation, participants verbally indicated which one of the three images they saw—A Fish, Truck, or Butterfly and the examiner clicked the corresponding keyboard arrow button (Fish = ←, Truck = ↓, Butterfly = →). Each child completed 32 trials which took approximately 5 min in duration. This psychophysical task has been used to reliably assess visual information processing (Brown and Crewther, 2017; Ebaid et al., 2017; Ebaid and Crewther, 2019).

##### Rapid Automatic Naming (RAN) as a Measure of Visual Object Verbalization

**Figure 2:**
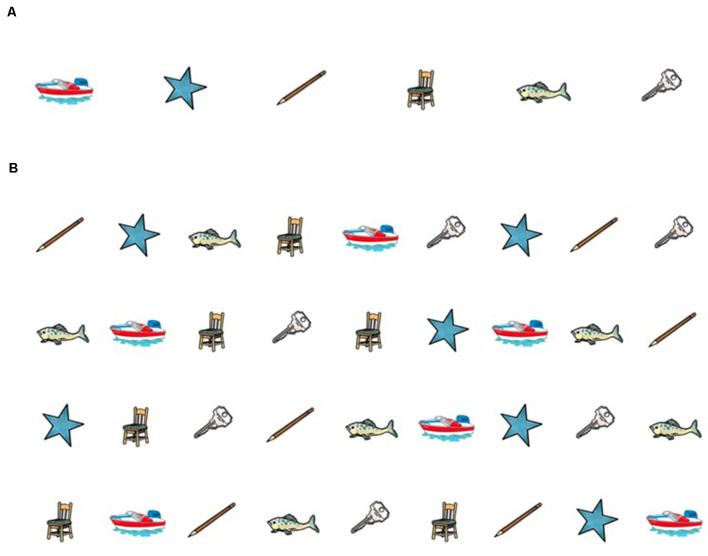
Visual object verbalization, rapid automatized naming (RAN). **(A)** Practice trial, **(B)** timed task.

The RAN of familiar objects task is a task requiring visual object verbalization. RAN has long been considered a predictor of reading ability (Denckla and Rudel, 1974; Georgiou et al., 2013; Siddaiah and Padakannaya, 2015). RAN has been regularly used in reading research since first introduced by Denckla and Rudel (1974) and is widely used in children aged 4–10 with test-retest reliability *r* = 0.77 (Denckla and Rudel, 1974; Wagner et al., 1999; Crewther et al., 2011, 2017; Savage et al., 2018; Barutchu et al., 2020; Peters et al., 2020). RAN of objects was chosen as a measure of how fast and accurately a participant could verbally name all 36 everyday objects shown on one A4 sheet. The task began with a practice trial using all objects (boat, star, pencil, chair, fish, and key), to ensure that each participant was familiar with all objects and the agreed name. The participants were then instructed to sequentially name, as quickly as possible, the series of nine objects in each of the four rows starting in the top left corner. The time taken to name all objects was recorded using a stopwatch (see Figure 2).

##### Slurp (Rate of Visuomotor Processing)

**Figure 3:**
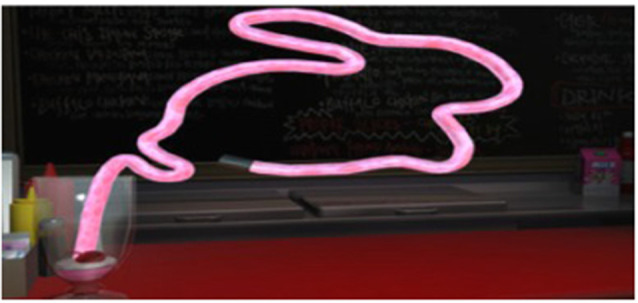
Example of Rabbit shape requiring visually guided tracing in the SLURP task.

The Lee-Ryan Eye-Hand Coordination Test (SLURP) was used to test the development of the visuomotor rate of information processing. SLURP is an iPad application developed by Lee et al. (2014) and designed by Malcolm Ryan to assess eye-hand coordination in terms of accuracy (number of errors) and time. This novel task has been demonstrated to be reliable and valid data and is normed for populations (5–88 years) across the lifespan (Junghans and Khuu, 2019). The task is game-like and requires children to trace shapes with their fingers as quickly and accurately as possible. The task begins with the Castle shape as a practice trial and then five shapes for the actual task in the following order (Circle, Tringle, Square, Rabbit and Snail). Slurp is a task that requires a motor response that involves vision and sustained visual attention to accomplish the task (see Figure 3). The total task duration is approximately 2 min.

#### Procedure

All testing sessions were conducted during school hours in a quiet room on school grounds. Each session was limited to 30 min maximum and varied according to the child’s attention span, interest, and motivation. Participants were asked which game they preferred to start with; the iPad (SLURP) or the computer game (IT). Short breaks were provided when requested and in between tasks. Reinforcements in the form of praise were provided to children at the end of each task.

#### Data Analysis

All data were analyzed using IBM SPSS statistics version 26. The data were checked for normality according to Shapiro-Wilk’s test (*p* > 0.05) with two outliers being identified and removed following inspection of boxplots. Pearson product-moment correlation analysis was used to explore the relationships between nonverbal IQ, chronological age CA, months at school (MAS) and the three experimental measures of rates of visual processing. For initial analysis assessing developmental changes in nonverbal IQ and cognitive processing task performance, participants were divided into three chronological age (CA) groups (5, 6 and 7 years; see demographics Table 1) and then into school Grades assuming this would differentiate children by 1 year of formal schooling (Prep, Grade 1 and Grade 2). Following assumption testing, one-way ANOVAs were conducted to determine whether performance differed significantly on the nonverbal IQ (RCPM), visual, visual-verbal processing tasks (IT and RAN) and the SLURP visuomotor across age groups and grade levels.

Hierarchical regressions were then conducted to investigate the degree to which the length of time attending formal school and presumably studying the domain-specific measures of visual-verbal information processing that is associated with reading and visuomotor (eye-hand co-ordination) processing as necessary for writing affects nonverbal IQ in children aged between 5 and 7 years. Measures of visual information processing were entered at step one to investigate each task’s specific contribution to predicting performance on the nonverbal IQ. Age and MAS were entered at step two to investigate the additional contribution to nonverbal IQ beyond visual, visual-verbal processing and visuomotor performance. Path analysis was then performed in order to further examine the hypothesis regarding the mediating effect of age-related development of visual processing speed on nonverbal IQ using the PROCESS SPSS Marco (Hayes, 2017). All analyses were conducted with an alpha of *p* < 0.05 level of significance.

## Results

### Relationships Between Age and School Years, With Nonverbal IQ, Visual, Visual-Verbal Processing, and Visuomotor Performance

Pearson correlations presented in Table 2 demonstrated that Age (years) and MAS were significantly negatively correlated with RAN and SLURP task duration, indicating that time required to complete the tasks decreased with age and months of schooling. The relationships between nonverbal IQ raw scores and total time to complete the visual-verbal and visual-motor task were both moderate and negative as accurate performance became faster in both visually driven RAN for objects (reading-like) and SLURP (writing-like) activities with less time being required for completion especially in terms of months at school. The relationships between SLURP and RAN were moderate and positive (*r* = 0.411).

**Table 2:** Correlation (Pearson’s r) between age, months at school, nonverbal intelligence, visual processing, visual-verbal processing, and visuomotor skills.

Measure	2.	3.	4.	5.	6.
1. Age	0.905**	0.650**	−0.163	−0.427**	−0.348**
2. Months at school	-	0.618**	−0.055	−0.510**	−0.385**
3. IQ	-	-	−0.102	−0.315*	−0.304**
4. IT	-	-	-	0.157	−0.195
5. RAN	-	-	-	-	0.411**
6. SLURP	-	-	-	-	-

Note. **p ≤ 0.01, *p ≤ 0.05. IQ raw scores, Raven’s Coloured Progressive Matrices task; IT, Inspection Time; RAN, Rapid Automatized Naming visual object verbalization; SLURP, total duration in seconds.

### Differences in Nonverbal IQ and Rate of Processing Task Performance Across Age and Grades

**Figure 4:**
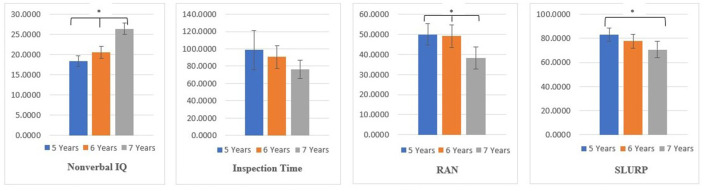
Age group differences (+/– 95% Confidence Intervals) for, nonverbal IQ, rate of visual information processing (Inspection Time), visual-verbal processing (RNA), and visuomotor processing (SLURP). Note. *p < 0.05.

To determine the age-related changes and the grades differences in nonverbal IQ (RCPM), visual processing (as measured by IT), visual-verbal processing (RAN), and visuomotor performance (SLURP) across the three age groups, 5 years, 6 years, and 7 years, a series of one-way ANOVAs were conducted (Figure 4). Group sizes, age and descriptive statistics for all dependent measures are shown in Table 3.

**Table 3:** Descriptive statistics for visual, visual/verbal, and visuomotor processing by age and grade.

		*M*	SD		*M*	SD
Nonverbal IQ (raw scores)	5 years	18.37	3.64	Prep	19.18	4.08
	6 years	20.54	4.54	Grade 1	24.89	4.53
	7 years	26.37	3.88	Grade 2	26.50	3.98
Inspection Time (ms)	5 years	98	60	Prep	91	51
	6 years	90	40	Grade 1	86	34
	7 years	76	30	Grade 2	70	14
RAN (Seconds)	5 years	49.96	12.03	Prep	51.61	12.27
	6 years	49.12	12.51	Grade 1	42.34	9.73
	7 years	38.19	9.55	Grade 2	38.19	8.74
Total duration SLURP (Seconds)	5 years	83.17	15.10	Prep	81.47	15.53
	6 years	77.73	17.27	Grade 1	70.36	15.66
	7 years	70.70	18.36	Grade 2	64.10	12.90

*ANOVA revealed significant differences between age groups for nonverbal IQ (*F*_(2.96)_ = 31.061, *p* < 0.000), RAN (*F*_(2.49)_ = 4.353, *p* < 0.018), and SLURP tasks (*F*_(2.94)_ = 4.071, *p* < 0.020), as scores in these measures increased significantly over time in years. *Post hoc* tests revealed that the 7-year-old group had significantly higher raw IQ scores and faster performance on the RAN task compared to the 5- and 6-year old age groups. Furthermore, the 7-year old group was significantly faster at completing the SLURP task compared to the 5-year old group. While the rate of visual processing appeared to decrease across age groups in the IT task, this change was not significant *F*_(2.90)_ = 1.946, *p* ≤ 0.149 (see Figure 4)*.

**Figure 5:**
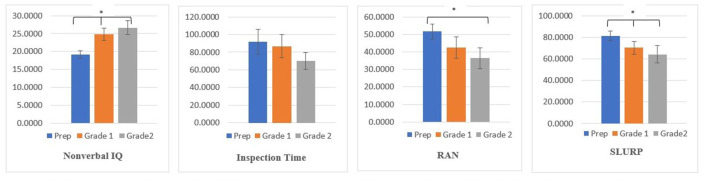
Grades means differences (+/– 95% Confidence Intervals) for, nonverbal IQ, rate of visual information processing (Inspection Time), visual-verbal processing (RNA), and visuomotor processing (SLURP). Note. *p ≤ 0.05.

The second ANOVA showed significant differences between the three grades for nonverbal IQ (*F*_(2.98)_ = 29.531, *p* < 0.000), RAN (*F*_(2.53)_ = 6.736, *p* < 0.002) and SLURP (*F*_(2.96)_ = 13.351, *p* < 0.000), as scores in these measures improved significantly with advancing grade level. *Post hoc* tests revealed that the Grade 1 and 2 students had significantly higher raw IQ scores and better performance on the Slurp task compared to the Prep. Further, the Grade 2 performed significantly in the RAN task compared to the Prep students (see Figure 5).

### Contribution of Visual Processing Speed, Age and MAS in Accounting for Nonverbal IQ

A series of multiple hierarchical regressions were conducted to determine how much each of the three visual domains (visual, visual-verbal and visuomotor) being investigated contribute to nonverbal IQ, and whether age or MAS contribute more to performance on the nonverbal IQ after controlling for task performance. The assumptions of multicollinearity, linearity, and homoscedasticity were not violated. Table 4 depicts the hierarchical regression outcomes for measures of speed of visual processing, age and MAS in predicting nonverbal IQ, respectively.

**Table 4:** Predictive contribution of visual, visual-verbal processing, and visuomotor skills, age and MAS on nonverbal IQ.

	Variable	Nonverbal IQ		
		*β*	*R*	sr
Step 1
	IT	−0.22	0.50	-0.21
	RNA	−0.34*	0.50	-0.29
	SLURP	−0.21	0.50	-0.18
		*R*^∧^^2^ = 0.20; *F* change _(3.44)_ = 4.848; *p* = 0.005		
Step 2	IT	−0.10	0.50	-0.098
	RAN	−0.11	0.50	-0.87
	SLURP	−0.11	0.50	-0.095
	Age	0.51*	0.65	0.424
			*R*^∧^^2^ = 0.38, Change *R*^2^ =0.18; *F* change _(1.43)_ = 13.516; *p* = 0.001 Total *R*^2^ = 0.43; *F*_(4.43)_ = 8.049	
Step 1	IT	−0.26	0.52	-0.18
	RAN	−0.39	0.52	-0.32
	SLURP	−0.17	0.52	-0.14
		*R*^∧^^2^ = 0.22; *F* change _(3.43)_ = 5.377; *p* = 0.003		
Step 2	IT	−0.19	0.52	-0.18
	RAN	−0.08	0.52	-0.061
	SLURP	−0.11	0.52	-0.090
		0.56*	0.69	0.454
			*R*^∧^^2^ = 0.43, Change *R*^2^ = 0.21; *F* change _(1.42)_ = 16.605; *p* = 0.000 Total *R*^2^ = 0.48; *F*_(4.42)_ = 9.648	

Note. IT, Inspection Time; RAN, Rapid Automatic Naming; SLURP, visuomotor performance task; Nonverbal IQ, Intelligence Quotient score on Raven’s Coloured Progressive Matrices task; MAS, Months at School. *p ≥ 0.01; according to Cohen’s guidelines, *r* ≥ 0.10, *r* ≥ 0.30, and *r* ≥ 0.50, represent small, medium, and large effect sizes, respectively (Cohen, 2013).

Regression analyses reveal that at the ages under investigation, visual processing measures and age significantly predict nonverbal IQ. The total contribution of visual processing tasks (IT, RAN, and SLURP) and age to predicting nonverbal IQ was 43%, with visual processing tasks accounting for 25% of the variance and age adding 18% of the variation. Examination of individual predictors revealed that RAN (visual-verbal processing) was the only significant predictor of nonverbal IQ in the first step, accounting for 8% of the variation whereas age in the second step accounted for 18%. Although chronological age was a significant predictor of nonverbal intelligence beyond visual processing measures, the second analysis demonstrates that MAS provides a further contribution to nonverbal IQ. Analysis of the contribution of visual processing speed tasks and MAS to nonverbal IQ was significant. The addition of MAS significantly accounted for a further 21% of variance beyond the contribution of visual processing measures (27% of variance), with both levels of the hierarchical regressions explaining 48% of the total variance in nonverbal IQ. The unique contribution of MAS to nonverbal IQ was larger than age, 20% and 18% respectively.

**Figure 6:**
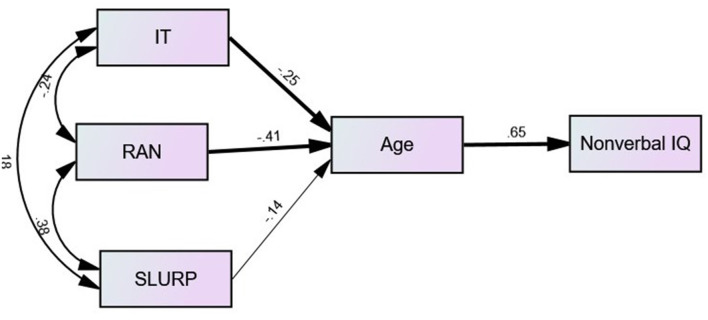
Path-diagram of age mediating the relationships between visual processing speed and nonverbal IQ. IT, Inspection Time; RAN, Rapid Automatic Naming; SLURP, visuomotor performance task; Nonverbal IQ, Intelligence Quotient score on Raven’s Coloured Progressive Matrices task. *Note*. Significant paths are in bold.

To further determine the mediating effect of age on processing speed and nonverbal IQ a path analysis was conducted (Figure 6). The regressions paths for both visual processing speed measures (IT and RAN) on age were significant (*b* = −0.25, *se* = 0.002, *p* = 0.017 and *b* = −0.41, *se* = 0.008, *p* = 0.001), respectively. However, the regression of visuomotor performance assessed with SLURP on age was not significant (*b* = −0.14; *se* = 0.005; *p* 0.197). The regression from the mediator (age) to nonverbal IQ was significant (*b* = −0.65; *se* = 0.509; *p* = 0.001). Based on 10,000 bootstrap samples (MacKinnon et al., 2004) a bias-corrected bootstrap confidence interval for the indirect effect of nonverbal IQ did not contain zero through age. The indirect effect of visual processing speed measures IT, RAN, and SLURP to nonverbal IQ through age was not significant (−0.16, −0.27, and −0.10), respectively.

## Discussion

This study aimed to investigate the contribution of age–related and school experience related differences in speed of information processing of visual, visual-verbal, visuomotor dual tasks and their relationships to nonverbal IQ of children aged 5–7 years old. We also aimed to examine the contribution of age and domain-specific school influences on the rate of processing to nonverbal IQ. The key findings were that nonverbal IQ was significantly correlated with age and MAS, and negatively correlated with the rate of sensory information processing (visual-verbal and visuomotor). Performance of the 7-year-old group showed a significant increase beyond that of the 5- and 6-years groups on measures of visual-verbal information processing assessed by RAN task, visuomotor skills tested by SLURP and nonverbal IQ, but not on simple visual information processing (IT task), for which there was a decreasing nonsignificant trend in threshold exposure time needed for accurate object identification. Grade 1 and 2 who attended formal schooling for 1 and 2 years longer than the Prep students, respectively, performed significantly better on RAN and SLURP tasks. Finally, MAS was associated with greater contributions to nonverbal intelligence than chronological age which supports the increases in domain-specific rate of processing.

### Relationships Between Nonverbal Intelligence and Sensory Information Processing

Consistent with our hypotheses and past research, nonverbal reasoning was significantly negatively correlated to the speed of visual-verbal and visuomotor information processing but not to simple visual perceptual task speed (Nettelbeck and Young, 1990; Coyle et al., 2011; Demetriou et al., 2014) with decreases in processing time being associated with increases in performance on nonverbal intelligence tests. Nettelbeck and Young (1990) examined the relationship between fluid intelligence (assessed on the Weschler Intelligence Scale for Children) and visual non-motor information processing speed in 6–7-year olds and found a moderate negative correlation (−0.31) similar to the findings of the current study. Furthermore, Kail (1991a) and Kail and Hall (1994) have also long observed that processing speed with motor components contributes significantly to intelligence. Indeed, Kail (2000) postulated that processing speed *per se* may be a great predictor of intelligence even in infancy. The efficiency of given information processing has also been noted to play a critical role in individual developmental differences of general intelligence (Demetriou et al., 2013). In terms of the visual perceptual and nonverbal IQ, the results show nonsignificant relationships between our IT task and nonverbal intelligence though a gradually increasing trend in performance of both which is in line with previous research investigating their relationship and suggesting insignificant and weak correlations especially in school age children (Smith and Stanley, 1983; Anderson, 1988, 1989; Mackenzie et al., 1991; Dandy, 2000). Many of these foregoing studies were included in a meta-analysis conducted by Grudnik and Kranzler (2001) and led to the conclusion that the association between IT tasks and IQ is comparable within the included studies. Jensen (2006) suggested a constant relationship between IT tasks and measures of IQ, which stands in contrast with our results even for a rank-order correlation between IT and nonverbal IQ that could be due to large variability in performance for our youngest children. Together, all these earlier studies in combination with the results presented here support the hypothesis that motor speed of information processing and fluid intelligence develop concurrently with age, and the corollary that fluid intelligence is usually accepted as a function of the rate of information processing.

### Visual, Visual-Verbal Information Processing and Its Relationship With Visuomotor Performance

Overall, our findings demonstrate a moderate and positive relationship (*r* = 0.356) between visual/verbal processing and visuomotor performance in children aged 5–7 years old while controlling for chronological age. Son and Meisels (2006) have previously reported a moderate positive relationship between cognitive skills and visual-motor skills in a longitudinal study of kindergarteners and Grade 1, *r* = 0.35 and *r* = 0.40, respectively. Further studies concur with our results, which present moderate to large associations between total score of cognitive abilities and fine motor performance in children aged 6–8 years old (Abdelkarim et al., 2017), and 4–11 years old (Davis et al., 2011). Our findings also lend support to previous literature indicating that cognitive and motor skills develop along the same timeline in children aged 5–10 years, though the strength of the relationship is less stable beyond 10 years of age (Anderson et al., 2001). Collectively, these findings indicate that children who perform well on cognitive skill tasks (fluid intelligence, visual processing, response inhibition, attention and working memory), are also more likely to perform well on visuomotor tasks (van der Fels et al., 2015). This significant association between both dual visually-driven motor measured functions (cognitive and fine motor) could be explained neuroanatomically as they share similar Magnocellular-driven attention and dorsal brain networks (Crewther et al., 1999; Laycock et al., 2007) and regions of interest (Leisman et al., 2016). Indeed the maturation of the dorsal visual stream (Goodale and Milner, 1992) and the dorsal streams’ dorsal and ventral goal-directed parieto-frontal pathways are thought to be responsible for visually driven attention action and goal directed behaviors and working memory (Corbetta and Shulman, 2002; Rizzo et al., 2017).

Simple visual information processing speed assessed with the IT task tended to increase with age but not at a rate that significantly correlated with visuomotor performance assessed by SLURP. This is in line with previous results by Ebaid et al. (2017) who investigated the relationship between visuomotor integration using Pegboard and processing speed measures (IT) in young and older adults and also found no significant relationships.

### Age/Grades and Performance on Nonverbal IQ and Measures of Visual Information Processing

As hypothesized, nonverbal IQ raw scores significantly increased with age and years of schooling, and the rate of visual-verbal processing assessed by RAN and visuomotor performance tested by SLURP decreased with age and length of time attending school. Our findings indicate that visually assessed nonverbal IQ and visual sensory information processing (visual-verbal and visual-motor) develop concurrently as children mature and become faster in processing and responding to sensory information, which in turn improves their reasoning capacities as evidenced by performance on tests of nonverbal intelligence such as RCPM (Fry and Hale, 2000; Kail, 2000; Cotton et al., 2005).

Visual-verbal processing and visuomotor performance are anecdotally reported to change significantly in children 5–7 years old when they begin formal schooling and start formally practising and utilizing their reading out loud and writing skills. Our findings also demonstrate that threshold IT continues to trend downward with age, though not to the point of reaching a significant level among the three age groups (5, 6, and 7-year-olds). This may be attributable to structural immaturity of the visual pathway projections (Hendrickson and Drucker, 1992; Crewther et al., 1999) that are not fully developed until late childhood/early adolescent years (Crewther et al., 1999; Hendrickson et al., 2012), in accordance with the functional development of the magnocellular (M) fast and parvocellular (P) slower projections to the visual cortex (Klistorner et al., 1997; Leat et al., 2009). Again, this is most likely because IT would be expected to be related to morphological maturation of the fovea of the retina around 5–6 years of age (Hendrickson and Drucker, 1992). By comparison, speed of processing of more complex dual visual tasks that are partially dependent on verbal or manual motor reaction time would be expected to initiate higher cognitive demands (selection and inhibition) and be highly affected by chronological age early in life (Nettelbeck and Wilson, 1985; Anderson, 1989; Nettelbeck and Young, 1989; Anderson et al., 2001).

Findings from the current study are consistent with previous results that have utilized speeded dual motor component tasks (time measurements) namely; perceptual motor tasks, the Tapping task, the Pegboard task, (Kail, 1991b) naming speed tasks, the coding task from WISC (Kail and Hall, 1994), RAN (Neuhaus et al., 2001), and response time tasks (Miller and Vernon, 1997) in children and confirm significant age differences in these tasks among children aged 4–8 years (Cotton et al., 2005). Our results share a number of similarities with these studies indicating that response time decreases as age increases and that the rate of change in information processing is faster in childhood.

### Contributions of Months of Early Schooling to Rates of Visual Information Processing and Nonverbal IQ

Our analyses demonstrate a positive contribution of schooling to the rate of visual information processing namely visual-verbal and visuomotor performance which is a reflection of the experience and practice of reading and writing once the child enters a formalized schooling system. This result is in line with Alexander and Martin (2004) who investigated the effect of schooling on cognitive abilities and suggested a greater influence of schooling than chronological age on verbal processing tasks, that are associated with reading ability. fMRI studies investigating the role of schooling 5–7 years children on brain function have demonstrated that practise and experience play a key role in brain activation, especially in the right posterior parietal cortex, that is associated with control of eye movements and shifts in attention (Wurtz and Goldberg, 1972), and executive function improvement (Burrage et al., 2008; Brod et al., 2017; Morrison et al., 2019). Similarly, Morrison et al. (2019), who reviewed the “casual” impact of schooling on cognition in school beginners (Pre-kindergarten, kindergarten, and Grade 1) demonstrated a strong impact on a variety of cognitive processing skills (attention control and working memory) that are essential for successful reading (visual-verbal) and writing (visuomotor integration). Our results are also in line with Duan et al. (2010) which highlight the more important role of knowledge and experience than age maturation in the speed of information processing development in children aged 9–13. Although age and time attending formal schooling are confounded, some studies have solved this issue by comparing two groups of students (Prep and Grade1) at the same chronological age, but where one group enrolled at school earlier than the other, and have also shown that 1 year of schooling has a stronger influence on cognitive functions namely, processing speed, sustained attention, working memory, cognitive flexibility, spatial ability, and inhibitory control than chronological age alone (Dasen et al., 2004; Burrage et al., 2008; Brod et al., 2017).

Our regression analyses supported the influence of domain-specific knowledge on visual processing development that is associated with practising of reading and writing abilities at schools. This related increase in the rate of visual-verbal (reading) and visuomotor performance (writing) due to formalized schooling significantly contributes to nonverbal IQ. Our results are in agreement with a wide range of studies that have compared the contribution of age and months of schooling effect to intellectual ability and suggested a greater contribution of schooling than age (Ceci, 1991; Artman et al., 2006; Cliffordson and Gustafsson, 2008; Brinch and Galloway, 2012; Ritchie et al., 2013). According to Cliffordson and Gustafsson (2008), months attending formal schooling significantly contributed to children’s performance on general intelligence tasks not only specific knowledge abilities that improve with regular formalized practice at school. Lastly, these studies have highlighted the usefulness of RAN as an early correlate measure of nonverbal IQ, and as a well-established predictive measure of potential reading ability in preschool and early readers (Anthony et al., 2007; Furnes and Samuelsson, 2009; Fricke et al., 2016; Peters et al., 2020).

## Conclusions

Overall, this study has demonstrated that the acquisition of more complex visually based skills such as visual-verbal and visual-motor information processing and nonverbal IQ, develop concurrently during the early school years. Visual-verbal and visuomotor processing correlated, significantly though simple visual processing assessed with thresholds IT task did not reach significance level with any variables. Regression analyses comparing the prediction of age vs. MAS beyond domain-specific rates of processing to nonverbal IQ indicate a larger contribution of MAS than chronological age to nonverbal IQ. Hence, our results support the domain-specific hypothesis demonstrating that months attending formal school contribute significantly more to cognitive performance than age, i.e., reading and writing associated abilities improve rapidly with regular practice (Burrage et al., 2008; Brod et al., 2017; Morrison et al., 2019), rather than the rate of simple visual information processing *per se* that is well developed even when starting school (Klistorner et al., 1997; Leat et al., 2009). Most importantly, the findings of this study provide further evidence that measures of rates of information processing in RAN and the SLURP are suitable measures for early identification of children likely to score lower in nonverbal IQ tests and have difficulties learning to read. However, further work with a larger sample size needs to be performed to determine the developmental changes of the rate of sensory information processing across wider age groups.

## Data Availability Statement

The raw data supporting the conclusions of this article will be made available by the authors, without undue reservation.

## Ethics Statement

The studies involving human participants were reviewed and approved by the La Trobe University Human Ethics Committee, the Victorian Department of Education Human Ethics Committee, and the Victorian Catholic Schools Ethics Committee. Written informed consent to participate in this study was provided by the participants’ legal guardian/next of kin.

## Author Contributions

RA and SC designed this study, and interpreted the data with MM. Both RA and MM conducted the data analysis and all authors contributed to writing up of the study. RA was assisted in data collection by Ms. Hayley Pickering and Ms. Areej Alhamdan. All authors contributed to the article and approved the submitted version.

## Conflict of Interest

The authors declare that the research was conducted in the absence of any commercial or financial relationships that could be construed as a potential conflict of interest.

## Publisher’s Note

All claims expressed in this article are solely those of the authors and do not necessarily represent those of their affiliated organizations, or those of the publisher, the editors and the reviewers. Any product that may be evaluated in this article, or claim that may be made by its manufacturer, is not guaranteed or endorsed by the publisher.
